# Achieving Diversity Through Decentralization

**DOI:** 10.1007/s11606-024-08780-0

**Published:** 2024-05-01

**Authors:** Eli Y. Adashi, Peter W. Marks

**Affiliations:** 1 Warren Alpert School of Medicine, Brown University, Providence, RI USA; 2 Center for Biologics Evaluation and Research, US Food and Drug Administration, Silver Spring, MD USA

Until relatively recently, randomized clinical trials (RCTs) — as advanced by Sir Ronald Aylmer Fisher and the subject of his “*Principles of Experimental Design*” — remained the inviolate cornerstone for optimally validating the utility and safety of drugs, biological products, and devices.^1^ Traditionally, most RCTs were conducted through coordination by a single central research site at academic medical centers. However, the conduct of RCTs grew increasingly challenging during the SARS-CoV-2 pandemic due to quarantines, site closures, and travel limitations, among other challenges. Mindful of this new reality, as well as of recent advancements in digital health technologies, the US Food & Drug Administration (FDA) issued Guidance for Industry, Investigators, and Institutional Review Boards on the *Conduct of Clinical Trials of Medical Products during COVID-19 Public Health Emergency* on March 13, 2020.^2^ This action not only facilitated the practical execution of clinical trials during the pandemic, but also incorporated the diversity-enhancing potential of alternative models for the conduct of clinical trials.

More recently, on December 29, 2022, the FDA was tasked by the Food and Drug Omnibus Reform Act of 2022 (FDORA) to issue or revise “draft guidance with recommendations related to using decentralized clinical studies to support the development of drugs and devices.”^3^ FDORA, a component of the *Consolidated Appropriations Act, 2023* [Public Law No:117-328], defined decentralized clinical trials (DCTs) as “a clinical study in which some or all of the study-related activities occur at a location separate from the investigator's location.”^3^ The steps taken by the FDA to advance the cause of DCTs in response to FDORA as well as their potential implementation are delineated below.

As per Section 3606 of FDORA, it was the expectation of Congress that “not later than 1 year after the date of enactment of this Act” the FDA will “issue or revise draft guidance that includes recommendations to clarify and advance the use of decentralized clinical studies to support the development of drugs and devices.”^3^ The FDA guidance is to include “recommendations for how to advance the use of flexible and novel clinical trial designs and to help improve trial participant engagement, recruitment, enrollment, and retention of a meaningfully diverse clinical population, including with respect to race, ethnicity, age, sex, and geographic location, when appropriate.”^3^

On May 3, 2023, the FDA issued the *Draft Guidance for Industry, Investigators, and Other Stakeholders: Decentralized Clinical Trials for Drugs, Biological Products, and Devices* using the definition of a DCT defined in FDORA. In fully decentralized clinical trials, all activities take place at locations other than traditional trial sites such as the homes of trial participants or in local healthcare facilities. In contrast, hybrid varieties of DCTs may feature in-person visits by trial participants to traditional clinical trial sites. Other visits or activities may involve locations other than traditional clinical trial sites such as the participant’s home.^4^ The decentralized nature of the DCT notwithstanding, the principal investigator remains responsible for the conduct of the DCT and oversight of individuals delegated to perform trial-related activities. Moreover, the principal investigator is to ensure that appropriate informed consent is obtained, the investigational product is appropriately administered in accordance with the protocol, and other required safety and efficacy assessments are done with appropriate documentation.

With an eye towards enhancing convenience for study participants who might otherwise be required to travel to distant clinical and laboratory resources, DCTs make a special point of enabling the use of local healthcare providers and local clinical laboratory facilities and of telehealth and digital health technologies to remotely acquire data. Stated another way, DCT participants may engage either from their home or else from their own preferred location. DCT participation may address some of the challenges faced by individuals in the community who cannot take time away from work to participate in RCTs or who are unable to or cannot afford to travel to RCT sites.

DCTs, however, are not a panacea to address all the challenges of RCTs, but rather constitute another tool that may help facilitate the conduct of clinical trials providing data that may be complementary to those obtained in RCTs that more closely reflects use of a treatment in clinical practice in the community. Just as for RCTs, DCTs require thoughtful planning and appropriate financial support, particularly regarding implementation of assessments that can be conducted by local providers with relative ease. Streamlining the amount of clinical data to be collected in a clinical trial to that which is clearly required to answer the clinical question at issue is crucial to help facilitate trial enrollment, retention, and compliance. Use of data collected remotely from wearable devices may assist in streamlining data collection. However, this may bring with it its own challenges, such as access to a wireless network. Additionally, recruitment strategies need to be strategically implemented to avoid recapitulating enrollment biases that may occur in RCTs and to reach individuals who might otherwise not participate in clinical trials. This may involve education of community practitioners participating in clinical research regarding how to explain the importance of clinical trials in easily understandable terms.

With appropriate planning, however, perhaps the one of the most important consequences of the adoption of DCTs may be the enhancement of the racial and ethnic diversity of clinical trials, which is often difficult to attain with centralized RCTs. Viewed in this light, DCTs are consistent with the long-standing commitment of the FDA to achieving scientific accuracy and social justice through the assurance of clinical trial diversity.^5,6^ Among the reasons cited by minority populations for this shortcoming of centralized RCTs, sentiments such as mistrust, discrimination, and miscommunication feature prominently. Needing to travel across significant distances to academic trial sites constitutes yet another relative shortcoming of centralized RCTs. In contrast, DCTs take place at or near home at sites where individuals will feel more comfortable participating due to established community practitioner relationships. This will likely enhance inclusivity of older adults as well. By correcting some of the limitations of traditional RCTs, DCTs should allow data to be obtained that will better reflect how products perform in the real-world use setting. The ultimate beneficiaries of the broad implementation of DCTs are likely to be underrepresented populations and older adults. In other words, DCTs may help diversify recruitment to clinical trials of representative participant populations.

The objective of better inclusion of underrepresented populations is a necessary and laudable aim to strive to accomplish. Barring unforeseen circumstances, all indications are that DCTs will continue to evolve on their way to becoming another important tool for the conduct of clinical studies complementary to centralized RCTs. Viewed in this light, DCTs may have transitioned from a novelty to a necessity. In so doing, DCTs are likely to expand access to clinical trials, facilitate research on rare diseases, increase trial efficiency, as well as improve trial participant recruitment, enrollment, retention, and diversity. To the degree that one can predict the shape of things to come, one must assume that DCTs constitute a paradigm whose time has come, especially since by providing more broadly applicable results they may provide important complementary information to RCTs that may be conducted in the process of product development.
